# A dual fluorescence channel RAA-based CRISPR-Cas12a/Cas13a system for highly sensitive detection of *Gyrovirus galga1* and *Gyrovirus homsa1*

**DOI:** 10.1080/21505594.2025.2521012

**Published:** 2025-06-22

**Authors:** Dan Yu, Zhixun Xie, Yanfang Zhang, Zhiqin Xie, Qing Fan, Sisi Luo, Liji Xie, Meng Li, Tingting Zeng, Minxiu Zhang, Xiaofeng Li, You Wei, Aiqiong Wu, Lijun Wan

**Affiliations:** a GuangXi Key Laboratory of Veterinary Biotechnology, GuangXi Veterinary Research Institute, Nanning, China; b Key Laboratory of China (Guangxi)-ASEAN Cross-Border Animal Disease Prevention and Control, Ministry of Agriculture and Rural Affairs of China, Nanning, China

**Keywords:** *Gyrovirus galga1*, *Gyrovirus homsa1*, recombinase acid amplification, CRISPR–Cas12a, CRISPR–Cas13a, dual-channel detection

## Abstract

*Gyrovirus galga1* (GyG1) and *Gyrovirus homsa1* (GyH1) are the second and third most common gyroviruses identified, respectively, after chicken anaemia virus. They were first reported in 2011 and are currently prevalent worldwide. However, limited research on these pathogens and a lack of prevention and control strategies have necessitated the establishment of a rapid diagnostic technique to address new challenges in infectious diseases. Recombinase acid amplification (RAA) combined with CRISPR – Cas12a or CRISPR – Cas13a technology has major advantages for highly sensitive and rapid diagnosis. Specific targets can activate CRISPR–Cas trans-cleavage activity, resulting in non-specific cleavage of single-stranded DNA by the CRISPR – Cas12a complex and RNA cleavage by the CRISPR – Cas13a complex. In this study, for the first time, we combined RAA-based CRISPR – Cas12a and CRISPR – Cas13a systems for simultaneous differential diagnosis of GyG1 and GyH1 infection. The results showed that dual fluorescence channel RAA-based CRISPR – Cas12a/Cas13a technology could detect GyG1 and GyH1 within one hour, with a minimum detection limit of 1.5 copies of the target DNA standard/µL and no cross-reactivity with other avian pathogens. In addition, this method could be used for clinical detection, with the results exhibiting high consistency with those obtained by qPCR. These findings demonstrate that our RAA-based CRISPR – Cas12a/Cas13a dual-channel detection system can detect two different subtypes of gyrovirus in a sample with good specificity and high sensitivity, improving the detection efficiency and providing a new technique for the study of viral infection dynamics.

## Introduction

The newly discovered viruses *Gyrovirus galga1* (GyG1) and *Gyrovirus homsa1* (GyH1) are the second and third viruses, respectively, of the genus *Gyrovirus* (GyV) belonging to the Anelloviridae family to be discovered after chicken anaemia virus (CAV) [1,2]. These viruses are small, nonenveloped, single-stranded circular DNA viruses with a genome length of approximately 2.4 kbp and contain three partially overlapping open reading frames (ORFs), of which ORF1 encodes the skeleton protein VP2, ORF2 encodes the apoptotic protein VP3, and ORF3 encodes the capsid protein VP1 [3]. The nucleotide sequence similarities between GyG1 and CIAV VP1, VP2, and VP3 are 42%, 42%, and 30%, respectively, and those between GyH1 and CIAV VP1, VP2, and VP3 are 43%, 42%, and 29%, respectively; the nucleotide similarities between GyG1 and GyH1 VP1, VP2, and VP3 are 62%, 63%, and 55%, respectively [4,5]. GyG1 and GyH1 occur mainly in mixtures with other pathogens when infecting chicken flocks [6]. Infected chickens present symptoms such as bleeding, oedema, glandular stomach erosion, and head swelling, which can lead to high morbidity and high mortality [7–9]. Other animals that have been reported to be infected with GyG1 include humans, wild birds, ferrets, dogs, domestic cats, snakes and ticks [10–14], and animals infected with GyH1 include humans, wild birds, ferrets and domestic cats [15–19]. The host range of the two pathogens is similar, and infected people show different severities of diarrhoea symptoms [2,14], suggesting that the two new GyVs have cross-species transmission potential.

GyG1 and GyH1 infect mainly chickens. In 2011, Rijsewijk et al. identified the genome of a distant relative of CAV in serum samples of diseased chickens in Brazil and reported it as a new GyV named avian gyrovirus 2 (AGV2) [1]. In 2012, to identify the cause of diarrhoea in children with acute gastroenteritis, Phan et al. conducted a viral metagenomic analysis of 100 human faecal samples and identified the presence of a gyrovirus in the samples, the third member of the GyVs – Gyrovirus 3 (GyV3) [2]. Subsequently, GyG1 and GyH1 were detected in Chile, the United States, Italy, Hungary, France, Hong Kong, mainland China, Japan, Brazil, and the Netherlands [10,14,19–23]. In 2015, GyG1 was first detected in chicken feather samples from live poultry markets in China [23]. Since then, GyG1 has been detected in chicken flocks in Shandong, Henan, Jilin, Jiangxi, Zhejiang, Yunnan, Guangdong, Guangxi and other provinces, with a positive detection rate of 1.9 ~ 27.6% [23–25]. In 2017, the virus was first detected in the faeces of broiler chickens with infectious viral gonadal gastritis in China [26]. Since then, it has been detected in chicken flocks in Jiangsu, Henan, Jilin, Gansu, Zhejiang, Guangdong, Guangxi and other provinces, with positive detection rates ranging from 0.6% to 12.5% [27,28]. As of 2024, sixteen kinds of GyVs have been identified [6], and the International Committee on Taxonomy of Viruses (ICTV) has reclassified the Anelloviridae family. The original AGV2 was renamed GyG1, and GyV3 was renamed GyH1 [6].

To date, there are few reports on these two new GyVs. The direct involvement of these viruses in the aetiology of specific diseases and their prevalence and host diversity remain unclear, and no suitable culture system to isolate and identify these viruses has been developed; therefore, these viruses cannot be propagated in large quantities. Moreover, their pathogenicity remains largely unexplored. These unknowns have potential implications for and pose threats to public health and the poultry industry. Since the first discovery of new GyVs more than a decade ago, prevention and treatment measures for GyG1 and GyH1 have been lacking, including commercial vaccines and drug therapies. The first step in the prevention and control of viral infections is pathogen detection, which can be used to monitor viral infections in a timely manner and provides a basis for subsequent evaluation of prevalence and variation, as well as the effectiveness of vaccines and drug treatments.

Currently, traditional PCR-based detection remains the mainstream detection method; it has high specificity and is convenient for sequence analysis and enzyme digestion analysis. However, PCR requires thermocycling equipment and can be used to detect only one GyV at a time, which increases the time and labor costs of pathogen detection. Given the similarity of the aetiology and clinical manifestations of GyG1 and GyH1, we propose a dual-channel detection system for identifying and detecting these two viruses simultaneously. Recombinase acid amplification (RAA) is an isothermal amplification technology that has emerged in recent years [29]. The primer design is simple, and the target fragment can be rapidly amplified (20 min) at a constant temperature (37 °C-42 °C) [30]. In addition, the target specificity and nuclease cleavage activity of clustered regularly interspaced short palindromic repeats (CRISPR) and CRISPR-associated protein (Cas) systems allow CRISPR–Cas technology to achieve single-nucleotide specificity and attomolar sensitivity at the same constant temperature (37 °C-42 °C) [31,32]. However, when the sample target concentration is low, the number of activated Cas proteins is limited, and many detectable signals cannot be generated in a short period to meet the requirements of rapid detection. Therefore, in this study, a CRISPR–Cas system and RAA technology were combined to achieve enhanced specificity and sensitivity for the detection of GyG1 and GyH1. CRISPR – Cas12a and CRISPR – Cas13a are Class 2 effector-type microbial CRISPR–Cas systems [33]. The NUC lobes of Cas12a lack the HNH domain but contain the RuvC domain, which can cleave single-stranded DNA (ssDNA) indiscriminately once it is combined with a specific crRNA [34]. Cas13a is composed of two higher HEPN domains; once the CRISPR–Cas complex is formed, it cleaves single-stranded target and collateral RNAs in a non-specific manner [35]. We added CRISPR – Cas12a and CRISPR – Cas13a to the same reaction system and introduced a FAM-labelled ssDNA reporter and a ROX-labelled ssRNA reporter to achieve dual-channel detection of FAM and ROX fluorescence in one reaction tube, similar to duplex real-time PCR. The amplification curve was observed via the corresponding fluorescence channel with a fluorescence quantitative PCR instrument to achieve simultaneous identification and detection of GyG1 and GyH1, and an innovative, efficient RAA-based CRISPR – Cas12a/Cas13a dual-channel detection method was thus developed.

## Materials and methods

### Viruses and clinical samples

A GyG1-positive liver sample from a sick chicken with neurological symptoms, a GyH1-positive glandular stomach sample from a healthy chicken in a live poultry market, and a sample positive for mixed infection with GyG1 and GyH1 from the pooled visceral tissues of a chicken with tumor symptoms were collected and verified via PCR-based sequencing. The DNA materials of 12 closely related pathogens, including chicken infectious anaemia virus (CIAV), fowl adenovirus 4 (FAdV4), H9 subtype avian influenza virus (H9-AIV), Newcastle disease virus (NDV), avian infectious bronchitis virus (IBV), infectious bursal disease virus (IBDV), chicken parvovirus (ChPV), avian leukosis virus (ALV), chicken astrovirus (CAstV), avian nephritis virus (ANV), *Mycoplasma gallisepticum* (MG) and *Mycoplasma synoviae* (MS), were previously preserved in the laboratory and were used here to evaluate the specificity of the assay. A total of 192 clinical samples were collected from commercial chicken flocks in Guangxi, China, comprising three categories: throat/cloacal swabs (*n* = 116) from chickens; tissue samples (*n* = 48; including liver, bile, and glandular stomach); and environmental swabs (*n* = 28; including litter samples, sewage samples, and swab samples collected from the walls, floors and feed pads) obtained from feeding troughs and cage surfaces. All the samples were immediately frozen at − 80°C for preservation.

### Primer and crRNA design

#### Design and synthesis of primers and crRnas for GyG1

First, three crRNAs (named 12-crRNAs) were designed to screen out the most active crRNAs for the establishment of the CRISPR – Cas12a system. Conserved regions in GyG1 were identified through multisequence alignment using MegAlign, which revealed > 95% sequence conservation across 58 clinical isolates in the selected regions (Figure S1). Protospacer adjacent motifs (PAMs; 5”-TTTN-3,” where *N* = A/C/G) were selected based on their conservation and strategic positioning 20–25 bp upstream of target regions. Subsequently, the 20-bp sequences downstream of these PAMs were designated as crRNA spacers. Importantly, the first 8 nucleotides of these spacers constitute a highly conserved ‘seed region,’ critical for ensuring target specificity and minimizing off-target effects in the CRISPR-Cas12a system [36,37]. Experimental validation through in vitro cleavage assays and plasmid interference models confirmed that this seed region conservation governs Cas12a’s DNA recognition and cleavage efficiency [38]. Doudna’s research group previously obtained the optimal direct repeat sequence (5”-UAAUUUCUACUAAGUGUAGAU-3”) [39], and we formed an integral crRNA by integrating the spacer sequence with the 5’ direct-repeat sequence. Then, three primers with sizes of 28–32 bp and amplicon sizes of approximately 300 bp (namely, the forward and reverse RAA primers) were designed to bind to the periphery of the crRNA, and the primer sequence was designed to avoid pairing with the crRNA sequence. Finally, a pair of primers that bind to either side of the amplicon was designed for the plasmid standard. All sequences are shown in Table 1.

**Table 1.** Sequences of primers, crRnas and reporter probes.

	GyG1 (Cas12a)		GyH1 (Cas13a)	
	Name	Sequence	Name	Sequence
PCR Primers	GyG1-F	ATCCAAATTGGTATCGGGTCA	GyH1-F	CACAGGGCGGGCAGATCATAT
	GyG1-R	GCATAAATTCTCGGAGGTTAA	GyH1-R	GTCTCCAGTGCCATCTTCCCC
RAA Forward primer	GyG1-F1	ACGGACCAGCTCGCCAGAGATCTACGTC	GyH1-F1	TAATACGACTCACTATAGGG GAGCCGGATCAACGGGCAAGAAACTTAA
	GyG1-F2	CCAGCTCGCCAGAGATCTACGTCGGCTT	GyH1-F2	TAATACGACTCACTATAGGG GCCCAGCGGAGCCGGATCAACGGGCAAG
	GyG1-F3	CAGCTCGCCAGAGATCTACGTCGGCTTC	GyH1-F3	TAATACGACTCACTATAGGG CAGCGGAGCCGGATCAACGGGCAAGAAA
RAA Reverse primer	GyG1-R1	TAGCCTTACCACATAGGAGCCCGGGTGT	GyH1-R1	CCCCGCAGTTGCAGATCGCGTCGTGAGT
	GyG1-R2	CATAGGAGCCCGGGTGTGGGTTGAAGAT	GyH1-R2	CGTCTGAAGCCCCCGCAGTTGCAGATCG
	GyG1-R3	TTACCACATAGGAGCCCGGGTGTGGGTT	GyH1-R3	GACAGTCCTGCTGCCTCCTGGAACCAGT
crRNA	12-crRNA-1	**UAAUUUCUACUAAGUGUAGAU** GCUUCUACCACAGAGGACGA	13-crRNA-1	**GGACCACCCCAAAAAUGAAGGGGACCAAAAC** GCACGACUCUCCCUACCUGA
	12-crRNA-2	**UAAUUUCUACUAAGUGUAGAU** GAUAUGCGCGUAGAAGAUCC	13-crRNA-2	**GGACCACCCCAAAAAUGAAGGGGACCAAAAC** GCAUCCAAUAAGUUCGUCGG
	12-crRNA-3	**UAAUUUCUACUAAGUGUAGAU** AUCGCCGCGUUAAGAGGAGG	13-crRNA-3	**GGACCACCCCAAAAAUGAAGGGGACCAAAAC** AUUACCGUAUCGCUUCCUGG
Probes	ssDNA Reporter	FAM-TTATT-BHQ1	ssRNA Reporter	ROX-UUUUUU-BHQ1

Underlining indicates the T7 RNA polymerase promoter sequence; the crRNA direct-repeat sequence of Cas12a or Cas13a is in bold.

#### Design and synthesis of primers and crRnas for GyH1

First, three crRNAs (named 13-crRNAs) were designed for the establishment of the CRISPR – Cas13a system. The conserved region (>98% identity across 15 clinical isolates) was selected in the same way as described above for GyG1 (Figure S2), and three 20 bp reverse complementary sequences were designed, avoiding G bases in the 3” flanking sequence. We formed an integral crRNA by adding a stem–loop structure (with the sequence 5‘-GATTTAGACTACCCCAAACGAAGGGGACTAAAAC-3’) upstream of the target region and appended a T7 RNA polymerase promoter sequence upstream of the stem–loop sequence such that the crRNA could be produced on the basis of DNA templates through in vitro transcription (IVT). Then, three primers with a size of 28–32 bp and an amplicon size of approximately 300 bp (namely, the forward and reverse RAA primers) were designed to bind to the periphery of the crRNA, and on the basis of the CRISPR – Cas13a detection system, the T7 RNA polymerase promoter sequence (5‘-TAATACGACTCACTATAGGG-3’) was added to the 5”-end of the forward primer. Finally, a primer pair that binds to either side of the amplicon was designed for the plasmid standard. All sequences are shown in Table 1.

### Genomic DNA extraction and plasmid standard synthesis

The collected swab and tissue samples were presoaked with PBS, squeezed or ground, and subjected to 3 cycles of freezing at −80°C and thawing. Two hundred microlitres of viral supernatant was added to the sample well of a 96-well plate from a prepacked viral RNA/DNA extraction kit (Biovet Biotech Co., Ltd., Tianjin, China), and the plate was placed in an NPA-32P automatic nucleic acid extraction and purification instrument (Bioer Technology Co., Ltd., Hangzhou, China). After 18 min, the genomic DNA of the sample was directly removed from the nucleic acid well and stored at − 20°C.

Three positive target DNA templates were amplified by using the primers GyG1-F/R and GyH1-F/R to prepare positive plasmid standards, and after the plasmid concentration was measured and calculated, the three plasmids were stored at − 20°C.

### Standard RAA and the CRISPR–Cas system

In the 50 µL RAA system, different primer sets were added to the detection unit tube according to the instructions of the basic RAA kit, and target sequence amplification was completed by incubation in a constant-temperature metal bath at 39 °C for 30 min. Among the 20 µL CRISPR–Cas systems, the GyG1 CRISPR–Cas12a system was configured according to the instructions for LbCas12a nuclease (EDE0005; Editgene Co., Ltd., Guangzhou, China), and the GyH1 CRISPR–Cas13a system was configured according to the instructions for LbuCas13a nuclease (EDE0002; Editgene Co., Ltd., Guangzhou, China) and the T7 transcription kit (JT101–01; Editgene Co., Ltd., Guangzhou, China). The resulting CRISPR–Cas system was incubated at room temperature for 15 min to form a CRISPR–Cas complex. Two microlitres of RAA product was added to the tube cap, centrifuged immediately, mixed well, and centrifuged again. The fluorescence was detected and measured every minute by a QuantStudio5 fluorescence quantitative PCR instrument (Thermo Fisher Scientific Co., Ltd., Massachusetts, USA) at 37 °C to screen optimal primers and crRNA for subsequent experiments.

### Optimization of the RAA-based CRISPR – Cas12a/Cas13a reaction

#### Optimization of the RAA-based CRISPR–Cas12a system for GyG1

With the GyG1 DNA as a positive template, we adjusted the primer concentration, 12-crRNA concentration, and ssDNA reporter concentration based on the standard RAA and CRISPR – Cas12a systems to achieve efficient RAA-based CRISPR – Cas12a reactions. The primer concentrations (200 nM, 300 nM, 400 nM, 500 nM, and 600 nM), crRNA concentrations (8.3 nM, 16.7 nM, 25 nM, 33 nM, 41.3 nM, 50 nM, and 58 nM), and ssDNA reporter concentrations (200 nM, 300 nM, 400 nM, 500 nM, and 600 nM) were changed in turn, and the ideal working concentration was selected according to amplification curves in the FAM channel.

#### Optimization of the RAA-based CRISPR–Cas13a system for GyH1

With the GyH1 DNA as a positive template, we adjusted the concentrations of the primers, Cas13a, 13-crRNA, RNA reporter and T7 RNA polymerase on the basis of the standard RAA and CRISPR – Cas13a systems to achieve efficient RAA-based CRISPR – Cas12a reactions. The primer concentrations (200 nM, 300 nM, 400 nM, 500 nM, and 600 nM), Cas13a concentrations (25 nM, 50 nM, 75 nM, 100 nM, and 125 nM), crRNA concentrations (50 nM, 75 nM, 100 nM, 125 nM, 150 nM, 175 nM, and 200 nM), RNA reporter concentrations (200 nM, 300 nM, 400 nM, 500 nM, and 600 nM) and T7 transcription enzyme volumes (0.3 µL, 0.4 µL, 0.5 µL, 0.6 µL, 0.7 µL, 0.8 µL, 0.9 µL, and 1.0 µL) were changed to select the ideal working concentration according to amplification curves in the ROX channel.

#### Optimization of the dual-channel RAA-based CRISPR – Cas12a/Cas13a system

GyG1 DNA, GyH1 DNA and mixed GyG1 and GyH1 DNA were used as the three templates. According to the optimization results for the RAA-based CRISPR – Cas12a and RAA-based CRISPR – Cas13a systems, two pairs of primers, GyG1-F/R and GyH1-F/R, could be used for amplification in the same RAA system. Two microlitres of the RAA product was subsequently used to optimize the CRISPR – Cas12a/Cas13a cleavage system. The components were adjusted according to the optimization system above so that the fluorescence intensities of the FAM channel and ROX channel were approximately equivalent, and the adjusted values were used as the final optimized dual-channel system conditions for subsequent experiments.

### Specificity and sensitivity evaluation

The specificity of the reaction was evaluated by detecting other emerging infectious gastrointestinal pathogens associated with GyG1 and GyH1 and other common avian pathogens, including CIAV, FAdV4, H9-AIV, NDV, IBV, IBDV, ChPV, ALV, ANV, CAstV, MG, and MS. The results were based on the fluorescence values measured in the FAM and ROX channels via a fluorescence quantitative PCR instrument (Thermo Fisher Scientific Co., Ltd., Massachusetts, USA).

The target GyG1 standard and target GyH1 standard were subjected to tenfold serial dilution and thoroughly mixed. We subsequently evaluated the sensitivity using mixed positive plasmids over a continuous concentration gradient from 3.0 × 10° to 3.0 × 10^5^ copies/µL as templates, with ddH_2_O used as a negative control. The optimized RAA-based CRISPR – Cas12a/Cas13a reaction was performed, and the fluorescence was collected in the FAM and ROX channels to test the minimum detection limit.

### Application in clinical samples

To assess the reliability of our proposed dual fluorescence channel RAA-based CRISPR – Cas12a/Cas13a diagnostic system, 192 clinical samples collected in the laboratory were blinded during testing, with parallel testing against PCR and qPCR methods [40]. All PCR-positive and qPCR-positive amplicons were subjected to Sanger sequencing verification (Sangon Biotech, Shanghai, China) to exclude false-positive results. Statistical analysis was performed via SPSS 26.0 software to calculate Cohen’s κ coefficient (two-tailed significance threshold α = 0.05), and the agreement rate was calculated as follows: $\mathrm{Agreementrate}=\frac{\mathrm{Number}\;\mathrm{of}\;\mathrm{concordant}\;\mathrm{cases}}{\mathrm{Total}\;\mathrm{number}\;\mathrm{of}\;\mathrm{cases}}=\frac{a+d}{N}$

where:

- α = Number of cases where both methods classify as positive (true positives).
- *d* = Number of cases where both methods classify as negative (true negatives).

## Results

### Study protocol

**Figure 1.**
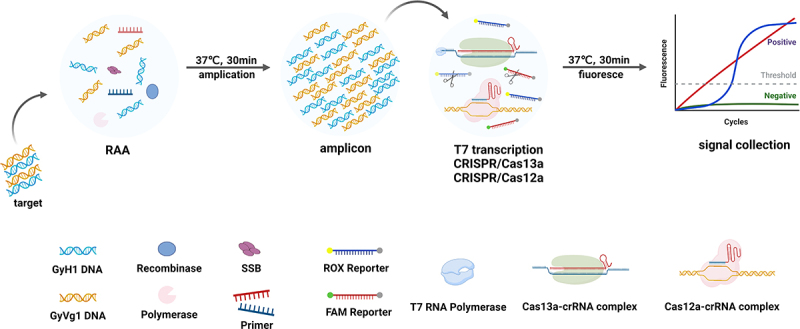
Workflow of the dual fluorescence channel RAA-based CRISPR – Cas12a/Cas13a diagnostic system.

First, the GyG1 and GyH1 targets were amplified at 37 °C for 30 min via RAA to obtain a sufficient target concentration. Second, crRNA, cas12a, and cas13a were incubated at room temperature for 15 minutes to form the Cas12a – crRNA complex and Cas13a – crRNA complex (when the RAA was running). The T7 transcription enzyme and RAA product were subsequently added to the premix CRISPR–Cas system, followed by another incubation at 37 °C for 30 minutes to allow specific cleavage of the reporter probes. At this time, RAA – CRISPR – Cas12a cleaved the ssDNA reporter, and posttranscriptional RAA – CRISPR – Cas13a cleaved the RNA reporter, thereby completing signal amplification detection. Finally, the fluorescence signals in the FAM channel and the ROX channel were collected to achieve rapid simultaneous detection of GyG1 and GyH1 within one hour (Figure 1).

### Screening of crRnas and RAA primers

**Figure 2.**
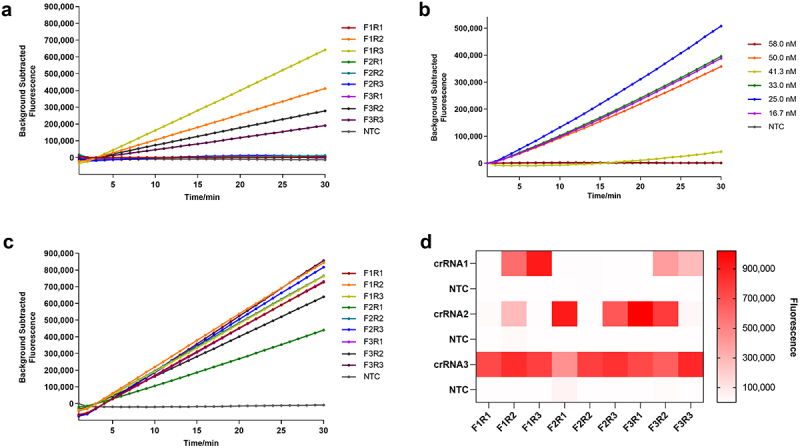
Screening results for the primers and crRnas for the RAA-based CRISPR – Cas12a method. (a) RAA primer combinations with crRNA1. (b) RAA primer combinations with crRNA2. (c) RAA primer combinations with crRNA3. (d) heatmap of the endpoint fluorescence values of 54 primer and crRNA combinations determined via RAA-based CRISPR – Cas12a.

**Figure 3.**
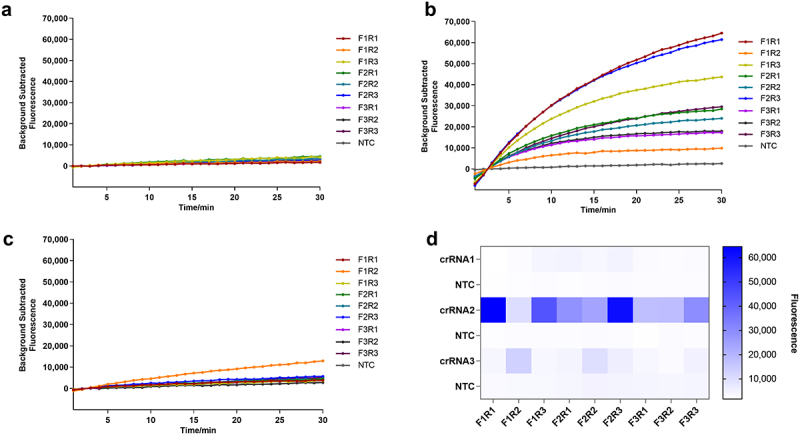
Screening results for the primers and crRnas for the RAA-based CRISPR – Cas13a method. (a) RAA primer combinations with crRNA1. (b) RAA primer combinations with crRNA2. (c) RAA primer combinations with crRNA3. (d) heatmap of the endpoint fluorescence values of 54 primer and crRNA combinations determined via RAA-based CRISPR – Cas13a.

Three upstream primers, three downstream primers, and three crRNAs were arranged in a total of 27 experimental groups, and 27 negative control groups were set up. The 54 combinations were simultaneously placed in a fluorescence quantitative PCR instrument, and the ideal crRNA and primers were screened according to the measured fluorescence. The results of the GyG1 primer and crRNA screening are shown in Figure 2. Using crRNA1 and crRNA2, only some experimental groups produced fluorescence (Figure 2a,b,d). Using crRNA3, all the experimental groups produced fluorescence, and the corresponding negative control group did not produce fluorescence (Figure 2c–d). On the basis of the curve appearance time and fluorescence intensity, we selected GyG1–F3, GyG1–R3 and crRNA3 as the ideal primers and crRNA. The results of the GyH1 primer and crRNA screening are shown in Figure 3. Neither crRNA1 nor crRNA3 produced fluorescence (Figure 3a,c,d). All the experimental groups with crRNA2 produced fluorescence signals, and the corresponding negative control group did not produce fluorescence signals (Figure 3b,d). Based on the curve appearance time and fluorescence intensity, we selected GyH1-F1 and GyH1-R1 as the optimal primers and crRNA2 as the optimal crRNA.

### Optimization of the RAA-based CRISPR – Cas12a/Cas13a reaction

#### Optimal reaction conditions for the RAA-based CRISPR – Cas12a system

**Figure 4.**
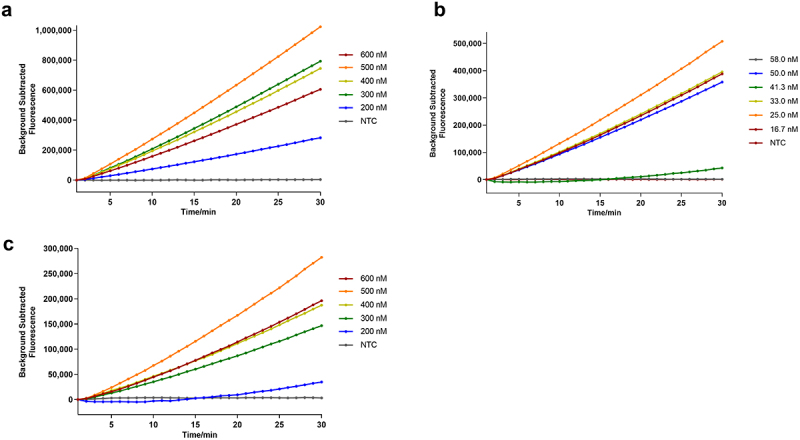
Results of optimization of the component concentrations for the RAA-based CRISPR – Cas12a system. (a) RAA primer concentration. (b) crRNA concentration. (c) FQ-ssDNA concentration.

As shown in Figure 4, when the primer concentration was 500 nM (Figure 4a), the crRNA concentration was 25 nM (Figure 4b), and the ssDNA reporter concentration was 500 nM (Figure 4c), the comprehensive reaction efficiency was the highest; that is, the optimal RAA-based CRISPR–Cas12a system was achieved: the first step (RAA) included buffer A (25 μL), the GyG1 primer (10 µM) (2.5 μL), the DNA template (5 µL), buffer B (2.5 µL), and ddH_2_O to 50 µL, and the second step (CRISPR–Cas12a reaction) included 10× cleavage buffer (2 µL), LbCas12a nuclease (1 µM) (1 µL), crRNA (500 nM) (1 µL), and the ssDNA reporter (4 µM) (2.5 µL). Finally, 2 µL of RAA product was added, and ddH_2_O was added to achieve a final volume of 20 µL.

#### Optimal reaction conditions for the RAA-based CRISPR – Cas13a system

**Figure 5.**
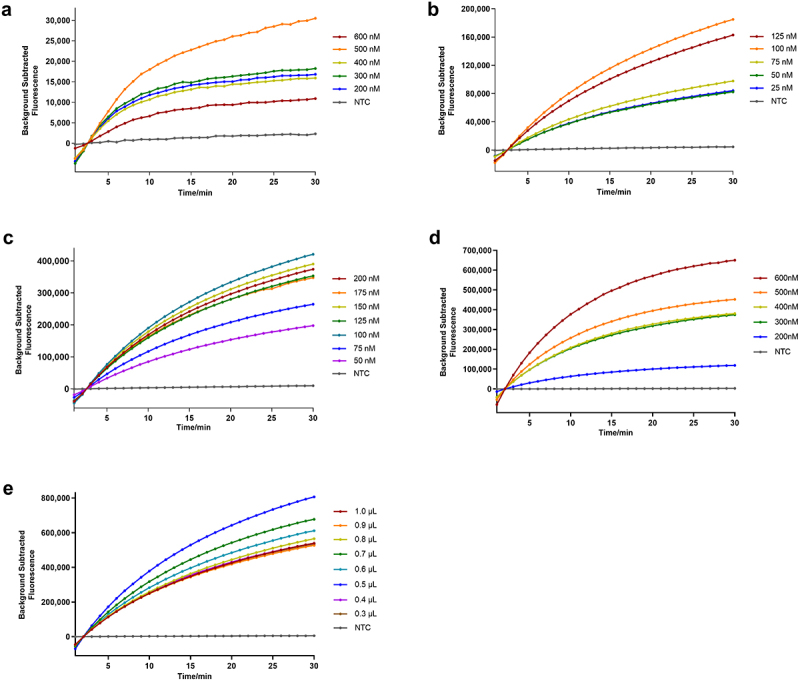
Results of optimization of the component concentrations for the RAA-based CRISPR – Cas13a system. (a) RAA primer concentration. (b) Cas13a concentration. (c) crRNA concentration. (d) RNA concentration. (e) T7 transcription enzyme volume.

As shown in Figure 5, when the primer concentration was 500 nM (Figure 5a), the Cas13a concentration was 100 nM (Figure 5b), the crRNA concentration was 100 nM (Figure 5c), the RNA reporter concentration was 600 nM (Figure 5d), and the T7 RNA polymerase volume was 0.5 µL (Figure 5e), the comprehensive reaction efficiency was the highest; that is, the optimal RAA-based CRISPR – Cas12a system was achieved: the first step (RAA) included buffer A (25 µL), GyH1 primer (10 µM) (2.5 µL), DNA template (5 µL), buffer B (2.5 µL), and ddH_2_O to 50 µL; the second step (CRISPR – Cas13a reaction) included 5 × cleavage buffer (4 µL), LbuCas13a nuclease (5 µM) (0.4 µL), crRNA (500 nM) (4 µL), RNA reporter (4 µM) (3 µL), 40 U RNase inhibitor (0.5 µL), NTP mix (0.8 µL), 5 × T7 transcription reaction buffer (1 µL) and T7 transcription enzyme mix (0.5 µL). Finally, 2 µL of RAA product was added, and ddH_2_O was added to achieve a final volume of 20 µL.

#### Optimal reaction conditions for the RAA-based CRISPR – Cas12a/Cas13a system

**Figure 6.**
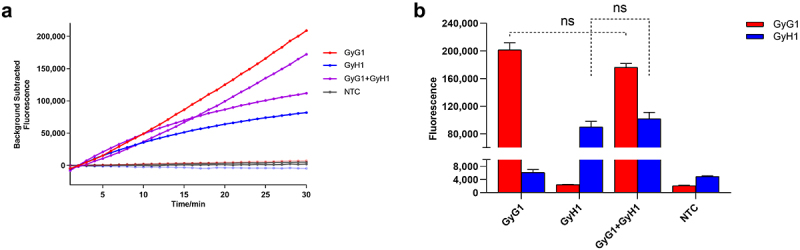
Results of optimization of the component concentrations for the RAA-based CRISPR – Cas12a/Cas13a system. (a) results from the QuantStudio5 fluorescence quantitative PCR instrument. (b) final fluorescence value at 30 min (*n* = 3 technical replicates; results are represented by mean ± sems; ***p < 0.001).

The proportions of each component of the CRISPR – Cas12a and CRISPR – Cas13a optimization systems were adjusted as a whole. When the GyG1-F/R and GyH1-F/R concentrations were 500 nM, the Cas12a concentration was 50 nM, the 12-crRNA concentration was 20 nM, the ssDNA reporter concentration was 400 nM, the Cas13a concentration was 100 nM, the 13-crRNA concentration was 100 nM, the RNA reporter concentration was 600 nM, the T7 transcription enzyme volume was 0.5 µL, and the fluorescence intensities collected in the FAM channel and ROX channel were comparable (Figure 6). The optimized dual-channel system is shown in Table 2, and this system was used for subsequent experiments.

**Table 2.** Optimal RAA-based CRISPR – Cas12a/Cas13a reaction system.

Component	Volume (µL)	Concentration
**First step: RAA reaction**		
A Buffer	25	
GyG1-F (10 µM)	2.5	500 nM
GyG1-R (10 µM)	2.5	500 nM
GyH1-F (10 µM)	2.5	500 nM
GyH1-R (10 µM)	2.5	500 nM
ddH_2_O	7.5	
Target	5	
B Buffer	2.5	
Total	50	
**Second step: CRISPR – Cas12a/Cas13a reaction**		
10 × cleavage buffer	2	1**×**
1 µM LbCas12a	1	50 nM
500 nM crRNA	0.8	20 nM
4 µM ssDNA reporter	2	400 nM
5 × cleavage buffer	4	1**×**
5 µM LbuCas13a	0.4	100 nM
1 µM crRNA	2	100 nM
4 µM RNA reporter	3	600 nM
40 U RNase inhibitor	0.5	1 U
A/G/C/UTP	0.2 each	1 mm each
5 × T7 transcription reaction buffer	1	0.4**×**
T7 transcription enzyme	0.5	
RAA product	2	
Total	20	

### Specificity and sensitivity of the RAA-based CRISPR – Cas12a/Cas13a system

**Figure 7.**
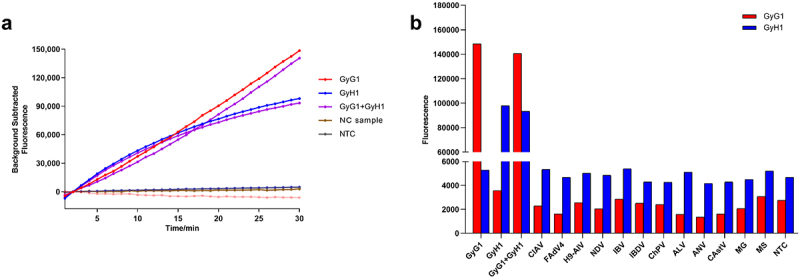
Specificity results for the dual fluorescence channel RAA-based CRISPR – Cas12a/Cas13a diagnostic system. (a) results from the QuantStudio5 fluorescence quantitative PCR instrument. (b) Final fluorescence value at 30 min.

The dual-channel RAA – CRISPR – Cas12a/Cas13a system was used to detect 15 reference strains under optimized conditions. GyG1 DNA, GyH1 DNA and mixed GyG1 and GyH1 DNA were used as three positive templates. Corresponding amplification reactions were performed for the three positive templates, which produced fluorescence amplification curves, whereas the other 12 avian pathogens (CIAV, FadV4, AIV-H9, NDV, IBV, IBDV, ARV, ALV, CAstV, ANV, MG, and MS) and ddH_2_O did not yield fluorescence signals (Figure 7). These results revealed that the two sets of primers and crRNAs designed in this study had good specificity, and the dual fluorescence channel RAA-based CRISPR – Cas12a/Cas13a system exhibited strong specificity.

**Figure 8.**
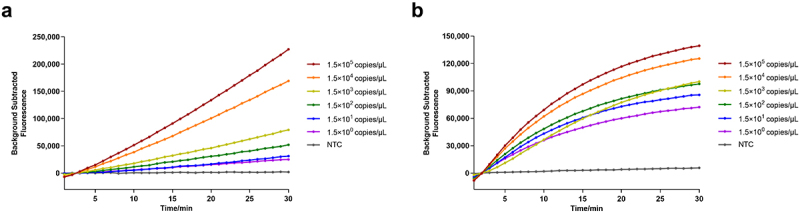
Sensitivity outcomes for the dual fluorescence channel RAA-based CRISPR – Cas12a/Cas13a diagnostic system. (a) Sensitivity outcomes for GyG1. (b) Sensitivity outcomes for GyH1.

The sensitivity was evaluated by using a mixture of 10-fold serial dilutions of the GyG1 and GyH1 DNA standards; that is, the actual working concentrations of the template were equivalent to 1.5 × 10° copies/µL to 1.5 × 10^5^ copies/µL. When the concentration of the GyG1 and GyH1 DNA standards was 1.5 copies/µL, the fluorescence signals in both the FAM channel and ROX channel could be detected (Figure 8a,b). Therefore, the detection limit of this diagnostic system was 1.5 copies/µL, indicating high sensitivity.

### Practical performance on clinical samples

To evaluate the diagnostic performance of the dual-channel RAA – CRISPR – Cas12a/Cas13a system, we analyzed 192 clinical samples, benchmarking the results against sequencing-validated PCR and qPCR as the reference standard. As shown in Table 3, the CRISPR assay identified 8 GyG1-positive (4.17%) and 6 GyH1-positive (3.13%) samples. The three methods demonstrated 100% concordance for samples with template quantities ≥  100 copies or 0 copies. However, in low-template samples (1–100 copies), the CRISPR assay detected one additional GyG1-positive sample (throat and cloacal swabs, *Ct* = 40) and one GyH1-positive sample (tissues, *Ct* = 39), both confirmed via postpurification Sanger sequencing to harbor GyG1- and GyH1-specific sequences. These results demonstrate the system’s enhanced sensitivity and specificity, effectively reducing the false-negative risk. Based on the qPCR standard curves (GyG1: Y = −3.321lgX +42.61; GyH1: Y= −3.231lgX +42.77), the copy numbers were calculated as 6.10 and 14.68, confirming the CRISPR method’s ability to detect low-copy targets in real-world samples.

**Table 3.** Comparative analysis of 192 clinical samples using RAA-based CRISPR-Cas12a/Cas13a, qPCR, and PCR assays.

Type of sample	Quantity of template (copies/µL)	GyG1			GyH1		
		qPCR	CRISPR (+)	PCR(+)	qPCR	CRISPR (+)	PCR(+)
Throat and cloacal swabs (*n* = 116)	≥100	2	2	2	0	0	0
	1~100	1	2	0	0	0	0
	0	113	0	0	116	0	0
Tissues (*n* = 48)	≥100	1	1	1	3	3	1
	1~100	0	0	0	1	2	0
	0	47	0	0	44	0	0
Environment samples (*n* = 28)	≥100	3	3	3	0	0	0
	1~100	0	0	0	1	1	0
	0	25	0	0	27	0	0
Total (*n* = 192)	≥100	6	6	6	3	3	1
	1~100	1	2	0	2	3	0
	0	185	0	0	187	0	0

In addition, statistical analysis revealed 98.44% concordance (κ = 0.79; 95% CI: 0.72–0.86) for GyG1 and 97.40% concordance (κ = 0.54; 95% CI: 0.41–0.60) for GyH1 between the two methods. The moderate GyH1 concordance likely reflects the limited statistical power due to the low number of confirmed positive qPCR results, which are compounded by potential inhibitors in clinical samples.

## Discussion

GyG1 and GyH1 are two novel gyroviruses that infect a wide range of hosts, including humans, and represent a risk to public health; they are distributed almost worldwide and cause coinfections, resulting in economic losses to the poultry industry. In addition, there are few studies and reports on GyG1 and GyH1, leading to many unknown risks. Therefore, early and rapid diagnosis is highly important for disease prevention and control, and molecular detection technology has played a major role in rapid diagnosis. CRISPR–Cas-based methods, known as “next-generation diagnostics [41]” and declared to be among the “Seven technologies to watch in 2022” [42], have been widely used in recent years. In 2017, the well-known HOLMES (Cas12a-based one-hour low-cost multiply highly efficient system) and DETECTR (DNA endonuclease-targeted CRISPR Trans Reporter) platforms [43,44] and the Cas13a-based SHERLOCK (specific high-sensitivity enzymatic reporter unlocking) platform were developed [45]. CRISPR-mediated diagnosis has broad application prospects in the next generation of diagnostic techniques, in-home diagnosis and point-of-care testing (POCT) [46–48].

As the “immune system” of microorganisms, the CRISPR system plays an effective role in spacer capture, crRNA maturation and cleavage of invading DNA. CRISPR – Cas12a binds to sufficient target to form a complex, exhibiting trans-cleavage activity at the target site and releasing fluorescence by cleaving the reporter probes, thereby facilitating diagnosis [49]. The methodological principle of CRISPR – Cas13, a diagnostic technology, is similar to that of CRISPR – Cas12a, as it recognizes RNA reporter probes, but an additional in vitro transcription process is needed [50]. Therefore, when the CRISPR – Cas12a and CRISPR – Cas13a systems are combined, upon the addition of a specific target, ssDNA reporter and RNA reporter, the target – LbCas12a – crRNA complex and target – LwuCas13a – crRNA are activated to cleave the reporter, and the results are displayed under the FAM channel and ROX channel, respectively. To obtain sufficient targets for the CRISPR–Cas system, we used RAA technology, which has high amplification efficiency and a short reaction time, to meet the need for prior amplification. Cas12a/Cas13a is responsible for the specific identification of enzyme-digested target sequences, and reporter probes are needed to visualize the detection results, which greatly improves the detection sensitivity and specificity.

The conserved regions of GyG1 and GyH1 were selected in the overlapping region of the VP2 and VP3 genes. Specific RAA primers and crRNAs were designed and screened to ensure the specificity of target cleavage. Following optimization of the RAA-based CRISPR – Cas12a and Cas13a systems, we systematically combined them at defined ratios to establish a dual fluorescence-channel RAA – CRISPR/Cas12a-Cas13a detection platform. This integrated system achieved simultaneous co-amplification of GyG1 and GyH1 targets, with fluorescence signals from mixed-target reactions demonstrating intensities comparable to those of single-target controls, confirming negligible primer-primer or enzyme-enzyme inhibition in the RAA – Cas12a/Cas13a integration system. Additionally, the inherent limitations of multiplex detection can be mitigated by calibrating the spectral alignment to reduce emission overlap between the FAM (520 nm) and ROX (610 nm) channels [51] while simultaneously implementing no-template controls (NTCs) and single-dye reaction controls to validate experimental outcomes. Previously, our laboratory developed a real-time fluorescence RAA-based detection method for GyG1 and GyH1. At that time, we combined the two fluorescence RAA methods for dual-fluorescence RAA-based detection. However, the results revealed strong fluorescence inhibition between the two channels, and subsequent experiments could not be carried out, indicating that the introduction of RAA-based CRISPR–Cas technology played an important role in specificity and had anti-interference effects. In addition, the overall working temperature of our experiment was 37 °C, which ensured that all the enzymes could function normally.

Despite being identified in 2011, GyG1 and GyH1 have not been successfully propagated in primary or passaged cell cultures. This limitation prevents the purification of intact virions and the quantification of genomic DNA in clinical samples, undermining real-world applicability evaluations and requiring plasmid standards as calibrated references for sensitivity validation. Our platform outperformed PCR and qPCR, with a detection limit (LoD) of 1.5 copies/µL for both GyG1 and GyH1 via plasmid standards—180-fold lower than that of PCR (2.7 × 10^2^ copies/µL) and 5-fold lower than that of qPCR (7.5 copies/µL). Integrated RAA – CRISPR achieved rapid detection within 60 minutes, 3 times faster than PCR (3 hours) and 1.5 times faster than qPCR (90 minutes), while maintaining equivalent sensitivity thresholds. We subsequently tested the specificity, and the dual-channel RAA-based CRISPR – Cas12a/Cas13a detection method did not exhibit cross-reactivity for other nontarget nucleic acids.

In conclusion, our proposed dual-fluorescence-channel RAA-based CRISPR – Cas12a/Cas13a detection method outperforms existing CRISPR diagnostic platforms in three critical aspects: cost efficiency, processing speed, and field-deployable simplicity. As the first demonstration of coordinated Cas12a/Cas13a coupling, this dual-fluorescence readout design achieves single-reaction discrimination of two circular viruses with clinical-grade performance. Time compression and parallel detection capacity not only address multiplex diagnostic bottlenecks but also establish a modular CRISPR architecture adaptable for diverse pathogen panels through enzyme/reporter reconfiguration; it can sensitively and accurately identify GyG1 and GyH1 in clinical samples, reduce the time and labor costs of detection, and is suitable for rapid detection and diagnosis of GyG1 and GyH1 infection in practice.

## Supplementary Material

Supplementary File.docx

Figure S1.jpg

Figure S2.jpg

## Data Availability

The data that support the findings of this study are openly available at https://doi.org/10.6084/m9.figshare.28466984.v1.

## References

[1] A RF, H DS, F TT, et al. Discovery of a genome of a distant relative of chicken anemia virus reveals a new member of the genus gyrovirus. Arch Virol. 2011;156(6):1097–14. doi: 10.1007/s00705-011-0971-6 21442232

[2] G PT, Li L, G OM, et al. A third gyrovirus species in human faeces. J Gen Virol. 2012;93(Pt 6):1356–1361. doi: 10.1099/vir.0.041731-0 22422066 PMC3755516

[3] Kraberger S, Opriessnig T, Celer V, et al. Taxonomic updates for the genus gyrovirus (family Anelloviridae): recognition of several new members and establishment of species demarcation criteria. Arch Virol. 2021;166(10):2937–2942. doi: 10.1007/s00705-021-05194-9 34347169

[4] T GP, N PV, Sdiri-Loulizi K, et al. Divergent gyroviruses in the feces of Tunisian children. Virology. 2013;446(1–2):346–348. doi: 10.1016/j.virol.2013.08.020 24074598 PMC3804065

[5] Shulman LM, Davidson I. Viruses with circular single-stranded DNA genomes are Everywhere! Annu Rev Virol. 2017;4(1):159–180. doi: 10.1146/annurev-virology-101416-041953 28715975

[6] Yan T, Wang Z, Li R, et al. Gyrovirus: current status and challenge. Front Microbiol. 2024;15:1449814. doi: 10.3389/fmicb.2024.1449814 39220040 PMC11362077

[7] Yao S, Gao X, Tuo T, et al. Novel characteristics of the avian gyrovirus 2 genome. Sci Rep. 2017;7(1):41068. doi: 10.1038/srep41068 28198372 PMC5309784

[8] Li G, Zhou D, Zhao M, et al. Kinetic analysis of pathogenicity and tissue tropism of gyrovirus 3 in experimentally infected chickens. Vet Res. 2021;52(1):120. doi: 10.1186/s13567-021-00990-2 34526128 PMC8442313

[9] Abolnik C, Wandrag DB. Avian gyrovirus 2 and avirulent Newcastle disease virus coinfection in a chicken flock with neurologic symptoms and high mortalities. Avian Dis. 2014;58(1):90–94. doi: 10.1637/10657-090313-Reg.1 24758119

[10] Feher E, Pazar P, Kovacs E, et al. Molecular detection and characterization of human gyroviruses identified in the ferret fecal virome. Arch Virol. 2014;159(12):3401–3406. doi: 10.1007/s00705-014-2203-3 25119678 PMC7087032

[11] Wu Q, Xu X, Chen Q, et al. Genetic analysis of avian gyrovirus 2 variant-related gyrovirus detected in farmed King ratsnake (Elaphe carinata): the first report from China. Pathogens. 2019;8(4):185. doi: 10.3390/pathogens8040185 31614719 PMC6963503

[12] Yang Z, Zhang J, Yang S, et al. Virome analysis of ticks in a forest region of Liaoning, China: characterization of a novel hepe-like virus sequence. Virol J. 2021;18(1):163. doi: 10.1186/s12985-021-01632-x 34372876 PMC8351423

[13] Xu S, Man Y, Yu Z, et al. Molecular analysis of *Gyrovirus galga1* variants identified from the sera of dogs and cats in China. Vet Q. 2024;44(1):1–8. doi: 10.1080/01652176.2024.2338381 PMC11008310 38595267

[14] K CD, L PL, S CS, et al. Characterization of a novel gyrovirus in human stool and chicken meat. J Clin Virol. 2012;55(3):209–213. doi: 10.1016/j.jcv.2012.07.001 22824231 PMC3449218

[15] M AD, J FS, C RB, et al. Faecal virome analysis of wild animals from Brazil. Viruses. 2019;11(9):803. doi: 10.3390/v11090803 31480274 PMC6784175

[16] Zhang S, Yang J, Zhou D, et al. Development of a DAS-ELISA for *Gyrovirus Homsa1* prevalence survey in chickens and wild birds in China. Vet Sci. 2023;10(5):312. doi: 10.3390/vetsci10050312 37235395 PMC10224540

[17] Cibulski S, D ADL, H FDS, et al. A plate of viruses: viral metagenomics of supermarket chicken, pork and beef from Brazil. Virology. 2021;552:1–9. doi: 10.1016/j.virol.2020.09.005 33032031 PMC7521440

[18] F CJ, K TK, Chen H, et al. Cross-species transmission and emergence of novel viruses from birds. Curr Opin Virol. 2015;10:63–69. doi: 10.1016/j.coviro.2015.01.006 25644327 PMC7102742

[19] Feher E, Pazar P, Lengyel G, et al. Sequence and phylogenetic analysis identifies a putative novel gyrovirus 3 genotype in ferret feces. Virus Genes. 2015;50(1):137–141. doi: 10.1007/s11262-014-1128-y 25319533

[20] Maggi F, Macera L, Focosi D, et al. Human gyrovirus DNA in human blood, Italy. Emerg Infect Dis. 2012;18(6):956–959. doi: 10.3201/eid1806.120179 22608195 PMC3358173

[21] H DS, B KM, de Castro F L, et al. Variants of the recently discovered avian gyrovirus 2 are detected in southern Brazil and the Netherlands. Vet Microbiol. 2012;155(2–4):230–236. doi: 10.1016/j.vetmic.2011.09.021 22018524

[22] Biagini P, Bedarida S, Touinssi M, et al. Human gyrovirus in healthy blood donors, France. Emerg Infect Dis. 2013;19(6):1014–1015. doi: 10.3201/eid1906.130228 23735883 PMC3713844

[23] Ye J, Tian X, Xie Q, et al. Avian gyrovirus 2 DNA in fowl from live poultry markets and in healthy humans, China. Emerg Infect Dis. 2015;21(8):1486–1488. doi: 10.3201/eid2108.150203 26196944 PMC4517707

[24] Yao S, Tuo T, Gao X, et al. Avian gyrovirus 2 in poultry, China, 2015–2016. Emerg Microbes Infect. 2016;5(10):e112. doi: 10.1038/emi.2016.113 27780970 PMC5117733

[25] Zhang Z, Man Y, Xu X, et al. Genetic heterogeneity and potential recombination across hosts of gyrovirus galga1 in central and eastern China during 2021 to 2024. Poult Sci. 2024;103(11):104149. doi: 10.1016/j.psj.2024.104149 39154608 PMC11381743

[26] Li G, Yuan S, He M, et al. Emergence of gyrovirus 3 in commercial broiler chickens with transmissible viral proventriculitis. Transbound Emerg Dis. 2018;65(5):1170–1174. doi: 10.1111/tbed.12927 29923685

[27] Yan T, Zhao M, Sun Y, et al. Molecular evolution analysis of three species gyroviruses in China from 2018 to 2019. Virus Res. 2023;326:199058. doi: 10.1016/j.virusres.2023.199058 36731631 PMC10194384

[28] Zhang S, Yuan S, Yan T, et al. Serological investigation of gyrovirus homsa1 infections in chickens in China. BMC Vet Res. 2022;18(1):231. doi: 10.1186/s12917-022-03334-0 35717195 PMC9206369

[29] Li J, Macdonald J, von Stetten F. Correction: review: a comprehensive summary of a decade development of the recombinase polymerase amplification. Analyst. 2020;145(5):1950–1960. doi: 10.1039/c9an90127b 31971531

[30] Tan M, Liao C, Liang L, et al. Recent advances in recombinase polymerase amplification: principle, advantages, disadvantages and applications. Front Cell Infect Microbiol. 2022;12:1019071. doi: 10.3389/fcimb.2022.1019071 36519130 PMC9742450

[31] Li H, Xie Y, Chen F, et al. Amplification-free CRISPR/Cas detection technology: challenges, strategies, and perspectives. Chem Soc Rev. 2023;52(1):361–382. doi: 10.1039/d2cs00594h 36533412

[32] Singh S, Raj D, Mathur A, et al. Current approaches in CRISPR-Cas systems for hereditary diseases. Prog Mol Biol Transl Sci. 2025;210:205–229. doi: 10.1016/bs.pmbts.2024.07.015 39824581

[33] Mohanraju P, S MK, Zetsche B, et al. Diverse evolutionary roots and mechanistic variations of the CRISPR-Cas systems. Science. 2016;353(6299):d5147. doi: 10.1126/science.aad5147 PMC13189112 27493190

[34] Strohkendl I, Saha A, Moy C, et al. Cas12a domain flexibility guides R-loop formation and forces RuvC resetting. Mol Cell. 2024;84(14):2717–2731. doi: 10.1016/j.molcel.2024.06.007 38955179 PMC11283365

[35] Liu L, Li X, Ma J, et al. The molecular architecture for RNA-Guided RNA cleavage by Cas13a. Cell. 2017;170(4):714–726. doi: 10.1016/j.cell.2017.06.050 28757251

[36] Zetsche B, S GJ, O AO, et al. Cpf1 is a single RNA-guided endonuclease of a class 2 CRISPR-Cas system. Cell. 2015;163(3):759–771. doi: 10.1016/j.cell.2015.09.038 26422227 PMC4638220

[37] Allen A, H CB, Singh J, et al. PAM-adjacent DNA flexibility tunes CRISPR-Cas12a off-target binding. Sci Rep. 2025;15(1):4930. doi: 10.1038/s41598-025-87565-9 39929897 PMC11811290

[38] Lu M, Yu C, Zhang Y, et al. Structure and genome editing of type I-B CRISPR-Cas. Nat Commun. 2024;15(1):4126. doi: 10.1038/s41467-024-48598-2 38750051 PMC11096372

[39] S CJ, Ma E, B HL, et al. CRISPR-Cas12a target binding unleashes indiscriminate single-stranded DNase activity. Science. 2021;371(6531):eabh0317. doi: 10.1126/science.abh0317 33602858

[40] Yu D, Xie ZX, Zhao JK, et al. Establishment of the duplex real-time PCR detection method for gyrovirus galga1 and gyrovirus homsa1. Chin J Vet Sci. 2025;45:59–65. doi: 10.16303/j.cnki.1005-4545.2025.01.09 (in Chinese.

[41] Chertow DS. Next-generation diagnostics with CRISPR. Science. 2018;360(6387):381–382. doi: 10.1126/science.aat4982 29700254

[42] Eisenstein M. Seven technologies to watch in 2022. Nature. 2022;601(7894):658–661. doi: 10.1038/d41586-022-00163-x 35079149

[43] S CJ, Ma E, B HL, et al. CRISPR-Cas12a target binding unleashes indiscriminate single-stranded DNase activity. Science. 2018;360(6387):436–439. doi: 10.1126/science.aar6245 29449511 PMC6628903

[44] Sinan S, M AN, W CC, et al. Kinetic dissection of pre-crRNA binding and processing by CRISPR-Cas12a. RNA. 2024;30(10):1345–1355. doi: 10.1261/rna.080088.124 39009379 PMC11404446

[45] S GJ, O AO, W LJ, et al. Nucleic acid detection with CRISPR-Cas13a/C2c2. Science. 2017;356(6336):438–442. doi: 10.1126/science.aam9321 28408723 PMC5526198

[46] Rathore P, Basnet A, Kilonzo-Nthenge A, et al. Rapid detection of pathogenic E. coli based on CRISPR Cas system. Front Microbiol. 2024;15:1423478. doi: 10.3389/fmicb.2024.1423478 38989031 PMC11233538

[47] S VR, Q KH, Koob J, et al. CRISPR-based rapid molecular diagnostic tests for fusion-driven leukemias. Blood. 2024;144(12):1290–1299. doi: 10.1182/blood.2023022908 38976877

[48] Tong X, Zhang K, Han Y, et al. Fast and sensitive CRISPR detection by minimized interference of target amplification. Nat Chem Biol. 2024;20(7):885–893. doi: 10.1038/s41589-023-01534-9 38332130

[49] A NE, M KR, Parikh I, et al. Determinants of CRISPR Cas12a nuclease activation by DNA and RNA targets. Nucleic Acids Res. 2024;52(8):4502–4522. doi: 10.1093/nar/gkae152 38477377 PMC11077072

[50] Jain I, Kolesnik M, Kuznedelov K, et al. tRNA anticodon cleavage by target-activated CRISPR-Cas13a effector. Sci Adv. 2024;10(17):l164. doi: 10.1126/sciadv.adl0164 PMC11042736 38657076

[51] Wijayanto AW, Murata T. Pre-emptive spectral graph protection strategies on multiplex social networks. Appl Netw Sci. 2018;3(1):5. doi: 10.1007/s41109-018-0061-8 30839797 PMC6214285

