# Supplementary material for: A dual fluorescence channel RAA-based CRISPR-Cas12a/Cas13a system for highly sensitive detection of Gyrovirus galga1 and Gyrovirus homsa1
Source: Virulence. 2025 Jun 22;16(1):2521012. doi: 10.1080/21505594.2025.2521012 (PMC12915420; doi:10.1080/21505594.2025.2521012)
Supplement: Supplementary File.docx [file KVIR_A_2521012_SM0509.docx]

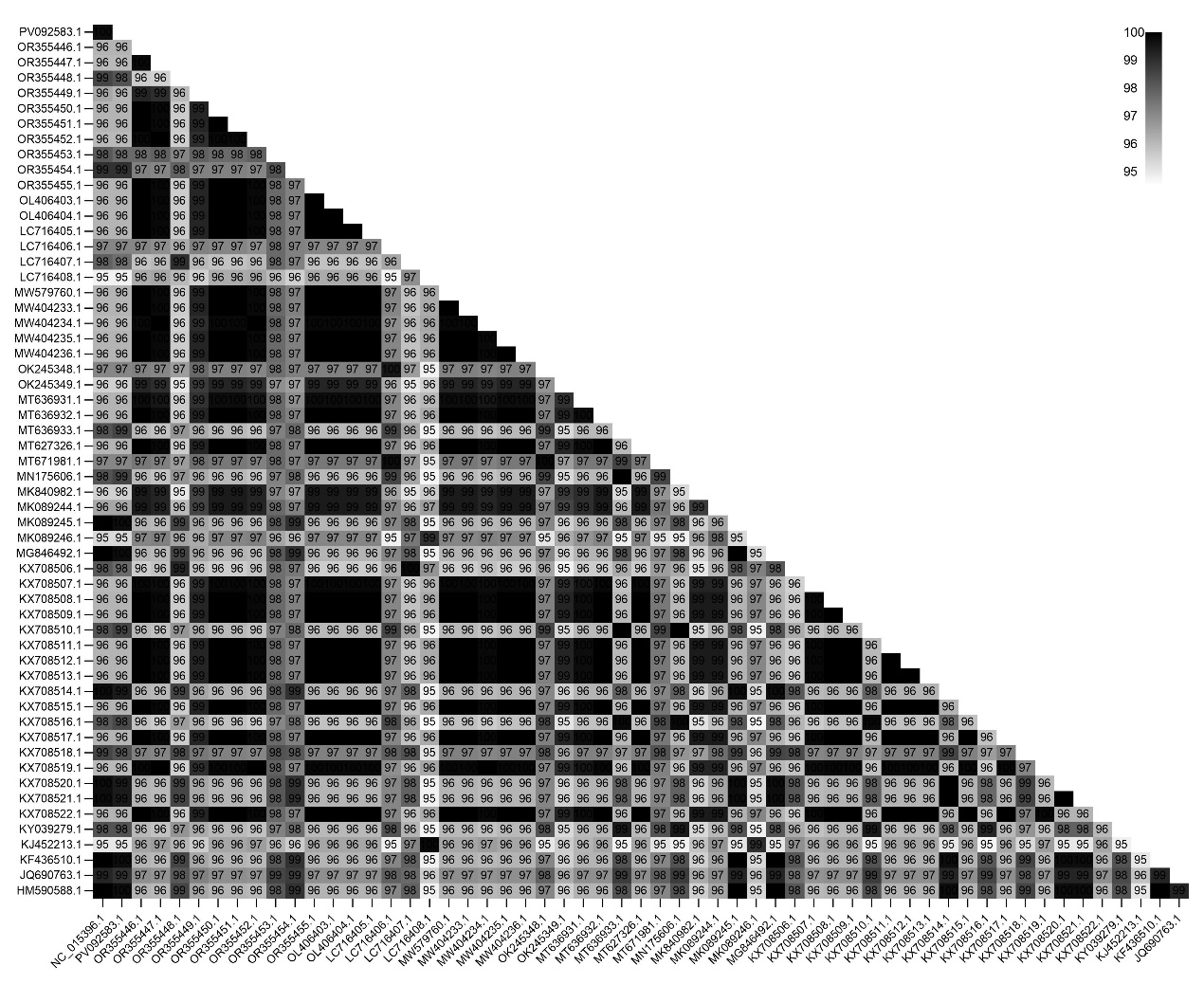


**Figure S1.** Similarity analysis of conserved regions of GyG1 (n = 58 strains) by multiple sequence alignment. (reference strain: NC_015396.1, 693-1314 nt)


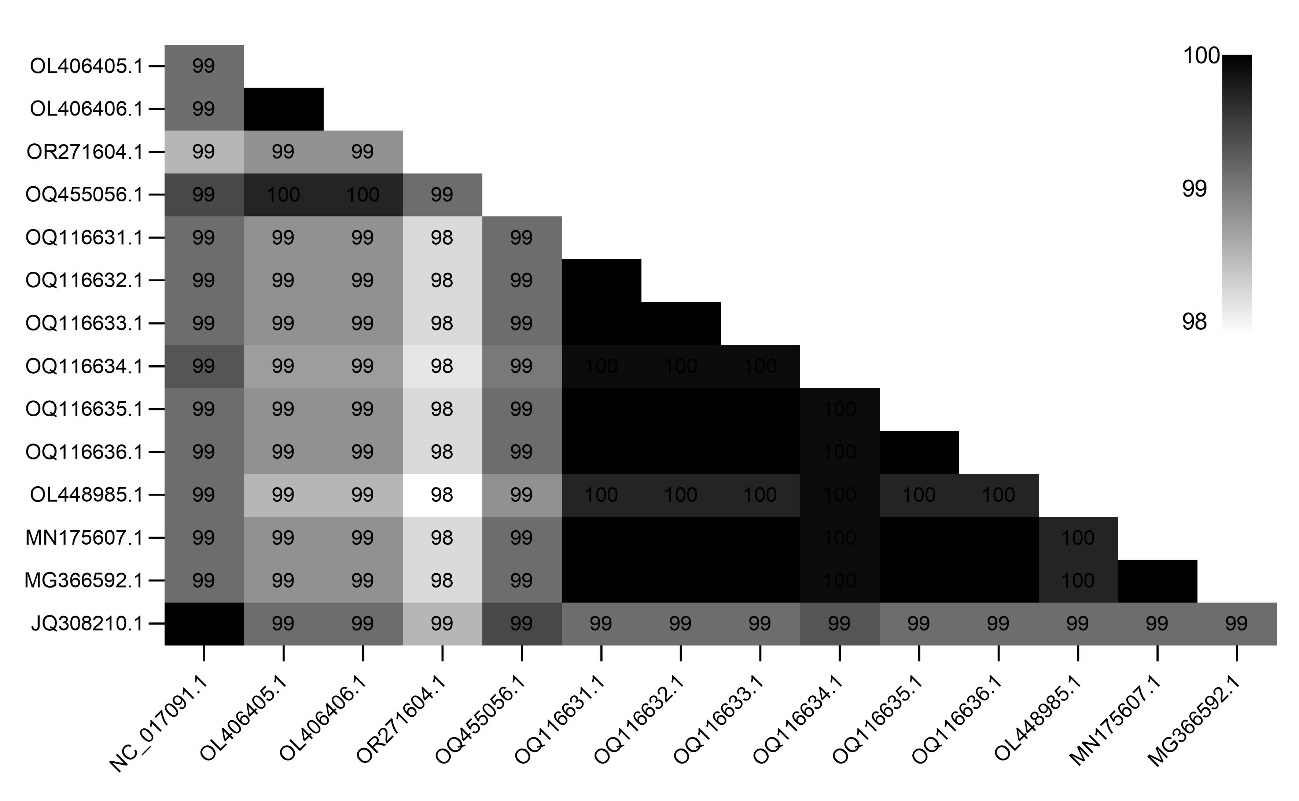


**Figure S2.** Similarity analysis of conserved regions of GyH1 (n = 15 strains) by multiple sequence alignment. (reference strain: NC_017091.1, 341-971 nt)
