# Supplementary figures and images for: A dual fluorescence channel RAA-based CRISPR-Cas12a/Cas13a system for highly sensitive detection of Gyrovirus galga1 and Gyrovirus homsa1
Source: Virulence. 2025 Jun 22;16(1):2521012. doi: 10.1080/21505594.2025.2521012 (PMC12915420; doi:10.1080/21505594.2025.2521012)

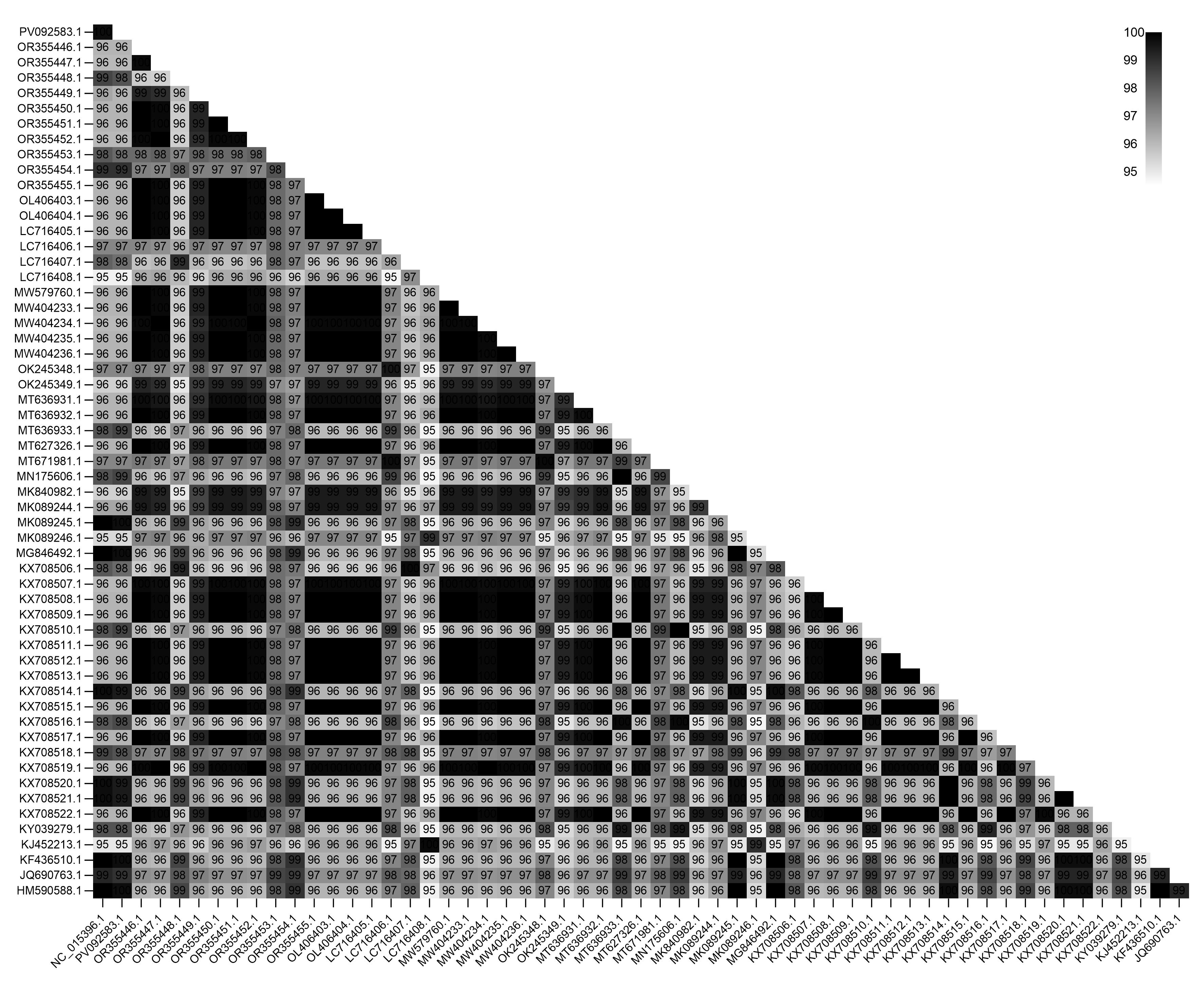

Supplement: Figure S1.jpg [file KVIR_A_2521012_SM0508.jpg]

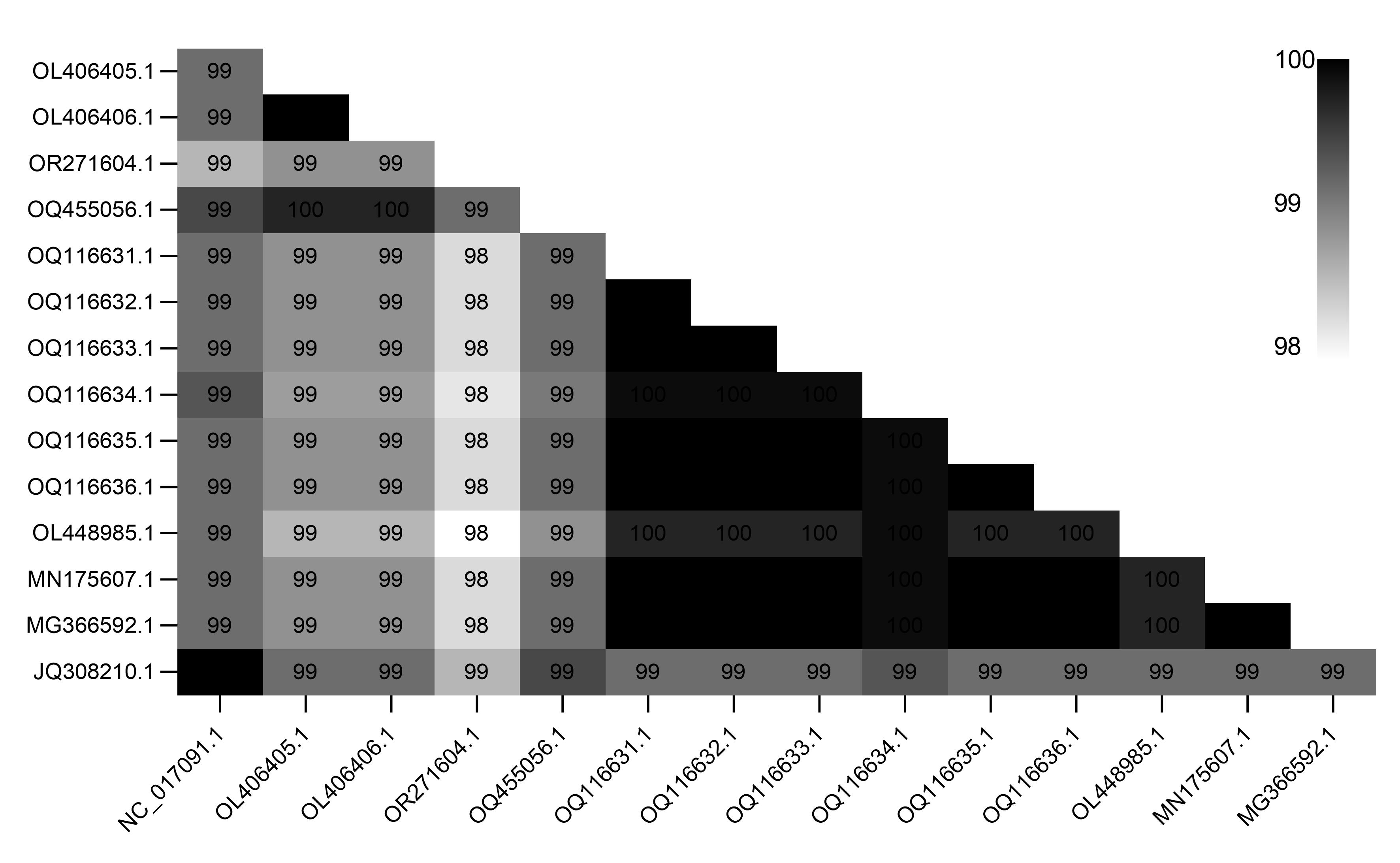

Supplement: Figure S2.jpg [file KVIR_A_2521012_SM0507.jpg]
